# pH-Dependent HEWL-AuNPs Interactions: Optical Study

**DOI:** 10.3390/molecules29010082

**Published:** 2023-12-22

**Authors:** Elena A. Molkova, Vladimir I. Pustovoy, Evgenia V. Stepanova, Irina V. Gorudko, Maxim E. Astashev, Alexander V. Simakin, Ruslan M. Sarimov, Sergey V. Gudkov

**Affiliations:** 1 Prokhorov General Physics Institute of the Russian Academy of Sciences, 119991 Moscow, Russia; bronkos627@gmail.com (E.A.M.); pustovoy@nsc.gpi.ru (V.I.P.); astashev@yandex.ru (M.E.A.); avsimakin@gmail.com (A.V.S.); rusa@kapella.gpi.ru (R.M.S.); 2 Physics Department, Belarusian State University, 220030 Minsk, Belarus; irinagorudko@gmail.com

**Keywords:** protein, nanoparticles, pH, hen egg-white lysozyme, nano-sized gold, aggregation

## Abstract

Optical methods (spectroscopy, spectrofluorometry, dynamic light scattering, and refractometry) were used to study the change in the state of hen egg-white lysozyme (HEWL), protein molecules, and gold nanoparticles (AuNPs) in aqueous colloids with changes in pH, and the interaction of protein molecules with nanoparticles was also studied. It was shown that changing pH may be the easiest way to control the protein corona on gold nanoparticles. In a colloid of nanoparticles, both in the presence and absence of protein, aggregation–deaggregation, and in a protein colloid, monomerization–dimerization–aggregation are the main processes when pH is changed. A specific point at pH 7.5, where a transition of the colloidal system from one state to another is observed, has been found using all the optical methods mentioned. It has been shown that gold nanoparticles can stabilize HEWL protein molecules at alkaline pH while maintaining enzymatic activity, which can be used in practice. The data obtained in this manuscript allow for the state of HEWL colloids and gold nanoparticles to be monitored using one or two simple and accessible optical methods.

## 1. Introduction

Advances in nanotechnology over the past few decades have made it possible to obtain gold nanoparticles with desired properties [1,2] for a wide range of biochemical and medical applications, including cancer therapy, drug delivery, biomarkers for medical imaging, and biological sensors [3,4,5]. Gold nanoparticles are attractive for biomedical applications due to their relative inertness [6]. Colloidal preparations of gold nanoparticles are often highly stable and may not precipitate for years. In addition, gold nanoparticles have important physical properties, such as surface plasmon resonance in the visible region, which has been successfully used in numerous test systems [7].

When nanoparticles enter biological fluids, their surface is often covered with a layer of biomolecules, mainly proteins, adsorbed on their surface [8]. Protein adsorption is a complex process involving interactions of different types, namely, van der Waals forces and electrostatic and hydrophobic interactions [9]. A dense layer of protein changes the size of the nanoparticles, which directly affects the properties of both the particle itself and the protein [10,11]. It is known that when nanoparticles enter the body, the cellular response may be associated with a reaction, not to the nanoparticle material itself but to the layer of protein molecules adsorbed on the surface and the change in secondary structure [12,13,14]. Changes in the properties of proteins upon interaction with nanoparticles are thought to depend on many factors, including the properties of the medium (pH, ionic strength, redox potential, etc.) [15].

Nowadays, the methods used to study the processes in colloids with nanoparticles and proteins often do not allow for the measurements in liquid (TEM) [16], require long-term measurements (electrophoresis) [17], which are invasive (viscometry) [18] and do not allow for the colloid to be studied repeatedly (most chemical methods), etc. [19,20]. The advantages of optical methods are known to be the speed of data acquisition, non-invasiveness, non-destructiveness, and the ability to obtain data over time. Disadvantages include limitations observed when working with large concentrations of proteins or nanoparticles. The aim of this study was to investigate the interaction of proteins and nanoparticles as a function of the pH of the environment over a wide range using basic optical methods available to most researchers. It has been shown that the use of even one or two available optical methods makes it possible to control the states of colloids containing protein molecules and nanoparticles. The results obtained in the manuscript may be in demand in the pharmaceutical industry, which produces protein preparations and test systems based on nanoparticles and enzymes.

## 2. Results

**Figure 1:**
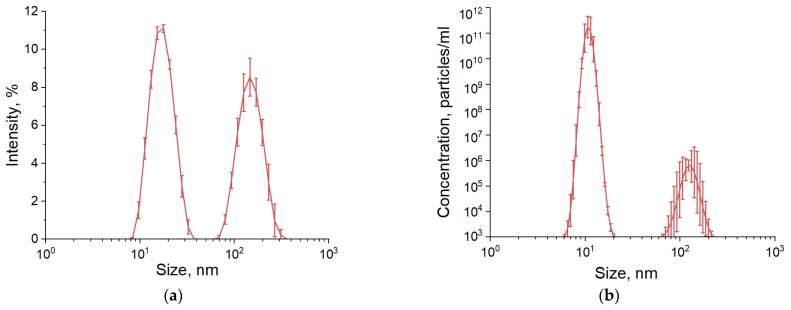

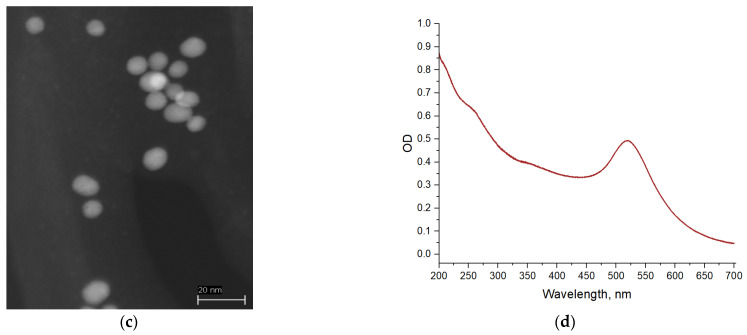
Physical characteristics of gold nanoparticles: (**a**) Size distribution of nanoparticles in an aqueous colloid; (**b**) Concentration of individual nanoparticles and their aggregates in the colloid; (**c**) TEM micrograph of gold nanoparticles; (**d**) Absorption spectrum of an aqueous colloid of gold nanoparticles.

**Figure A1:**
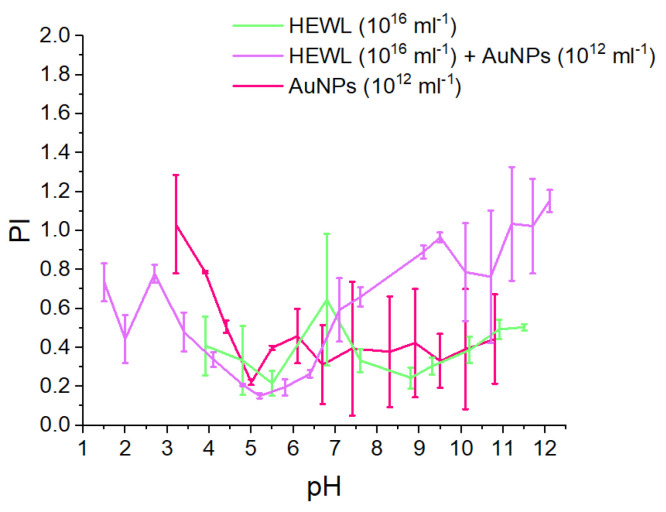
Effect of pH on the polydispersity index in colloids HEWL (7 × 10^4^ nM), AuNPs (2.2 nM), and HEWL (7 × 10^4^ nM) + AuNPs (2.2 nM).

**Figure A2:**
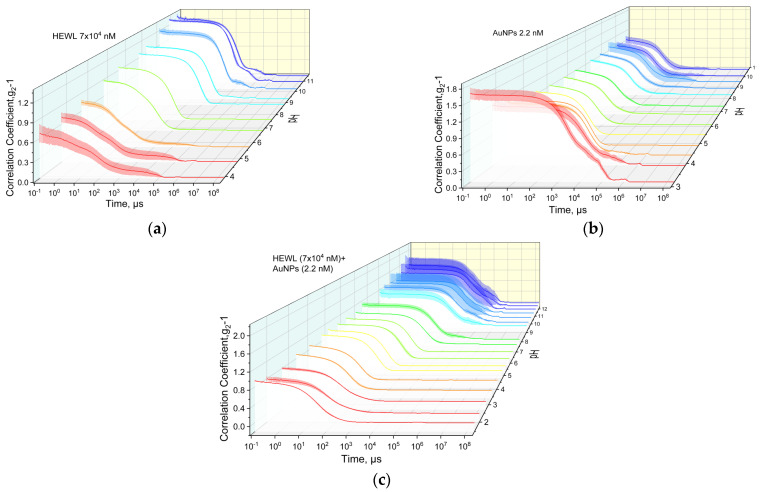
Autocorrelation functions of colloids: (**a**) HEWL, (7 × 10^4^ nM); (**b**) AuNPs (2.2 nM); (**c**) HEWL (7 × 10^4^ nM) + AuNPs (2.2 nM) at different pH values. The color of the lines has only an artistic component.

Gold nanoparticles were obtained using laser ablation in liquid. The DLS method was used to determine the size distribution of the gold nanoparticles. It was shown that the nanoparticle preparation has an average hydrodynamic diameter of 18 nm, and the width of the distribution at half-maximum intensity is 10–28 nm. In addition to individual nanoparticles, the colloid contains aggregates of nanoparticles formed together with single gold nanoparticles during laser ablation. The average hydrodynamic diameter of the aggregates is 150 nm, and the distribution width at half-maximum is 80–230 nm (Figure 1a). The Malvern Zetasizer Ultra was used to determine the concentration of individual nanoparticles and nanoparticle aggregates. The colloid was found to contain 1.3 × 10^12^ individual nanoparticles per ml of liquid. (Figure 1b). The concentration of aggregates was 3.1 × 10^5^; this means that for each aggregate, there were three million individual nanoparticles. The polydispersity index (PDI) can be used to assess the homogeneity of the system. Our colloid has a PDI of 0.3, from which we can conclude that the system behaves in a monodisperse manner (Figure A1). Also, the value of the autocorrelation function at the initial time is 0.9, which indicates the relevance of the data obtained (Figure A2b). The morphology of the nanoparticles was confirmed by transmission electron microscopy. It was shown that the nanoparticles have a spherical shape and a true size of the order of 12–15 nm (Figure 1c). In order to quickly determine the concentration of the nanoparticles, a spectroscopic study of the optical properties of the colloid of gold nanoparticles was carried out (Figure 1d). It was shown that the absorption spectrum contains an absorption maximum of 518 nm. To determine the stability of the nanoparticle colloid, ζ-potential measurements were performed. The ζ-potential of the gold nanoparticles was −26 mV.

**Figure 2:**
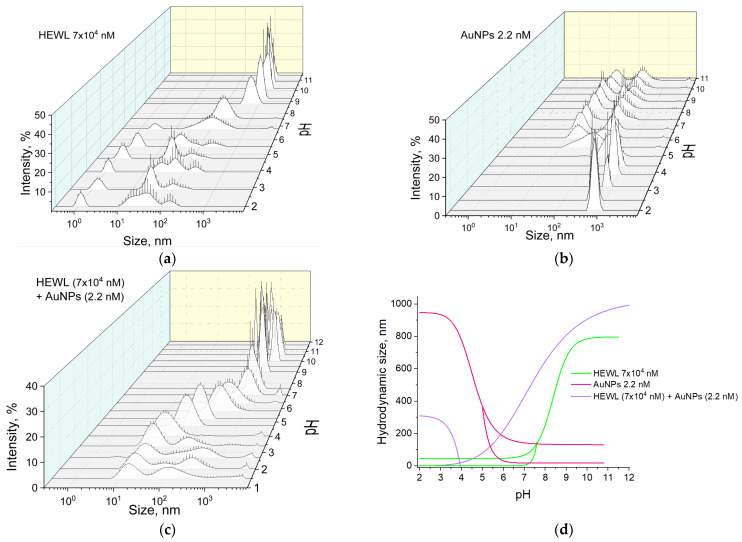
Effect of pH on the size distribution of objects in colloids: (**a**) HEWL, (7 × 10^4^ nM); (**b**) AuNPs (2.2 nM); (**c**) HEWL (7 × 10^4^ nM) + AuNPs (2.2 nM); (**d**) Dependence of the hydrodynamic diameter of objects in colloids HEWL (7 × 10^4^ nM), AuNPs (2.2 nM), and HEWL (7 × 10^4^ nM) + AuNPs (2.2 nM) on pH.

The effect of changes in pH on the size distributions in colloids of gold nanoparticles, HEWL molecules, and a colloid containing both gold nanoparticles and HEWL molecules was investigated (Figure 2a). When HEWL was dissolved in deionized water, the concentration was 7 × 10^4^ nM, and the pH of the colloid was 3.9. In this case, single HEWL molecules (size 1.2 nm) and aggregates of HEWL molecules with sizes from 15 to 300 nm are observed. There are approximately 3 × 10^8^ individual protein molecules per protein aggregate. When the pH of the solution is increased to 5.5, the size distribution of the HEWL preparation does not change significantly. When the pH of the colloid is increased to 6.8, the peak of individual HEWL molecules shifts toward larger sizes due to the dimerization of the molecules, and the size of the protein aggregates is located in a wider range of values from 20 to 400 nm. With further increase in pH, individual molecules and their dimers are not observed in the size distribution. The colloid contains only protein aggregates with an average size of about 650 nm or more. It is noteworthy that with increasing pH, compression of the aggregate sizes from 950 nm to 450 nm is observed; the average size is almost doubled with an increase in pH from 9.0 to 10.2.

The preparation of gold nanoparticles is obtained by the laser ablation process in deionized water with a concentration of 2.2 nM had a pH of 6.8 (Figure 2b). The average size of individual gold nanoparticles is 17–20 nm; aggregates of 80–160 nm were also observed in the colloid. When the pH is changed from 6.8 to 10.8, there is no significant change in the size of the nanoparticles and aggregates in the colloid. When the pH of the initial solution is reduced with HCl, the position corresponding to individual gold nanoparticles and the size distribution cannot be clearly identified. Only a broad peak of aggregates with sizes ranging from 20 to 300 nm is present. With further acidification of the environment, the size range of the observed aggregates decreases. At pH 3.2, two narrow peaks of nanoparticle aggregates with characteristic sizes of 300 nm and 750 nm are observed in the medium.

When gold and protein molecules are mixed, the size distribution shows a single peak with a maximum of 24 nm corresponding to gold nanoparticles surrounded by protein molecules, with a solution pH 4.0 (Figure 2c). There is no separate peak at 1–2 nm corresponding to individual protein molecules. As the pH decreases to 1.5, characteristic values of the hydrodynamic diameter of gold are observed, and the appearance of a small number of aggregates is also observed. At pH above 4.0, gold-coated HEWL molecules aggregate to form aggregates measuring 1 µm or more.

Figure 2d shows a general 2D size distribution graph for gold nanoparticles, HEWL molecules, and a colloid containing both gold nanoparticles and HEWL molecules. At pH 7.5 in the colloidal solution containing single molecules of HEWL and its aggregates, a sharp increase in the size of the aggregates from 100 to 800 nm occurs with the disappearance of the peaks of single protein molecules when moving to the alkaline pH region. The gold nanoparticle preparation is stable in the pH range = 6.0–11.0; changes in pH do not lead to changes in the size of individual molecules and aggregates (20 and 160 nm, respectively). When pH decreases to 3, only aggregates of gold nanoparticles with sizes of 300 nm and 700 nm are present in the solution. In the colloid containing both gold nanoparticles and HEWL molecules, two types of particles are observed in the pH range = 1.5–4.0. These are individual gold particles surrounded by HEWL molecules, approximately 24 nm in size, and aggregates of nanoparticles with protein, 350–100 nm in size. In this case, the peak corresponding to individual protein molecules is not observed. At pH = 6.0, only aggregated molecules of gold nanoparticles with HEWL size of 300 nm are present in the colloid, while the size of aggregates of gold nanoparticles in the stock solution was 160 nm and did not change with further alkalization of the medium. The size of the aggregates for the colloidal solution with gold nanoparticles and HEWL at pH = 7.5 was already 750 nm, which is five times larger compared to the HEWL stock solution (150 nm) at the same pH.

**Figure 3:**
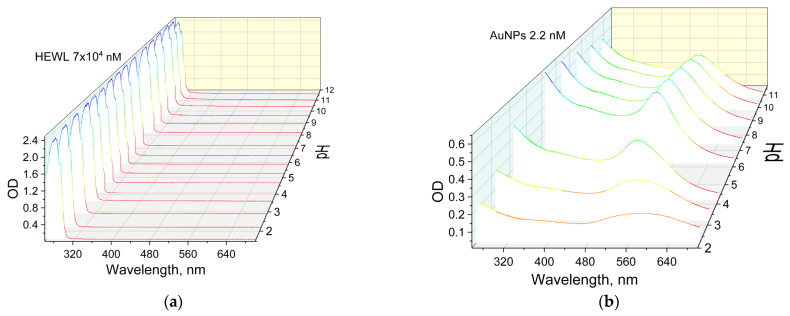

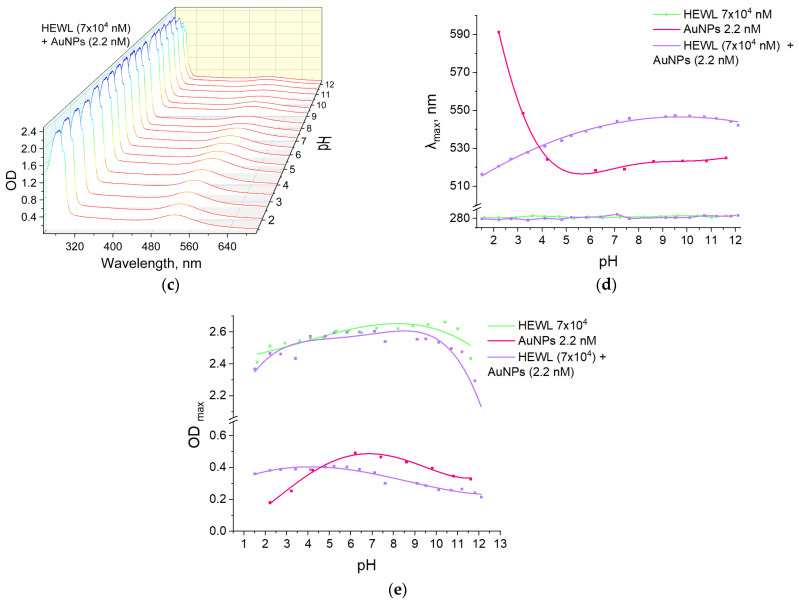
Effect of pH on the optical absorption of solutions: (**a**) HEWL (7 × 10^4^ nM); (**b**) AuNPs (2.2 nM); (**c**) HEWL (7 × 10^4^ nM) + AuNPs (2.2 nM); (**d**) Dependence of λ_max_ absorption on pH for colloids HEWL (7 × 10^4^ nM) + AuNPs (2.2 nM), HEWL (7 × 10^4^ nM), AuNPs (2.2 nM); (**e**) Dependence of optical density in maximum on pH for colloids HEWL (7 × 10^4^ nM) + AuNPs (2.2 nM), HEWL (7 × 10^4^ nM), and AuNPs (2.2 nM).

The effect of pH on the optical absorption of HEWL solutions was investigated (Figure 3a). It was shown that the maximum absorption of the HEWL stock solution is observed at 280 nm. When pH is changed, the position of the absorption maximum does not change; it is in the range of 280–281 nm. The absorption intensity in the whole range of measured pH values varies in the range of 2.5–2.6; only at points 1.5 and 11.6 do the OD (optical density) values decrease to 2.4. The effect of pH on the spectral characteristics of the colloidal solution of gold nanoparticles in the 450–700 nm wavelength range was investigated (Figure 3b). The maximum absorption of the colloid of gold nanoparticles at pH 6.8 is observed at the wavelength of 518 nm. When the pH is increased to 10.8, a slight shift in the position of the maximum towards the “red region” up to 525 nm is observed. When the pH is decreased from 6.8 to 2.2, a significant shift of the absorption maximum to the long wavelength region is observed at 590 nm.

The addition of protein molecules to the nanoparticles changes the pH dependence of the absorption maximum of gold nanoparticles (Figure 3c). At pH values from 1.5 to 4.1, the maximum absorption of gold nanoparticles is in the range of 521 to 524 nm. With a further increase in pH and transition to the alkaline region, the value of the absorption maximum shifts to 550 nm. This phenomenon confirms the correctness of the obtained dependencies of the size distributions of complexes of gold nanoparticles with proteins (Figure 2c).

When a colloidal system containing both gold nanoparticles and HEWL molecules is formed, no significant changes occur in the HEWL absorption region (280–281 nm). In the range of λ = 500–600 nm, the line profile of the pH dependence of λmax for gold nanoparticles mixed with HEWL changes symmetrically and inversely (Figure 3d). For pH values of 6.0–11.0, λmax for gold nanoparticles is in the range of 518–524 nm, but with decreasing pH, the value of the maximum wavelength shifts to larger values, namely, up to 590 nm at pH 2.2. When HEWL is added to gold nanoparticles, λmax takes values of 540–550 nm in the pH range = 6.0–11.0. When the medium is acidified, a shift toward the “blue region” to λ = 524 nm is observed; i.e., the process is opposite to that observed for the stock solution of gold nanoparticles. The two λmax curves of gold nanoparticle colloids and gold nanoparticles with the addition of HEWL intersect at pH = 4.2. At the same pH value, the ODmax curves for the same colloidal solutions intersect (Figure 3e). The optical density value for the solution of gold nanoparticles with HEWL decreases monotonically from 0.38 to 0.21 when the medium is basified from pH 1.5 to 12.1. The OD of the stock solution of gold nanoparticles decreases significantly from 0.49 to 0.18 when moving along the pH scale toward acidic values from pH = 6.2. This can be attributed to the formation of aggregates and the increase in the aggregate size of the gold nanoparticles.

**Figure 4:**
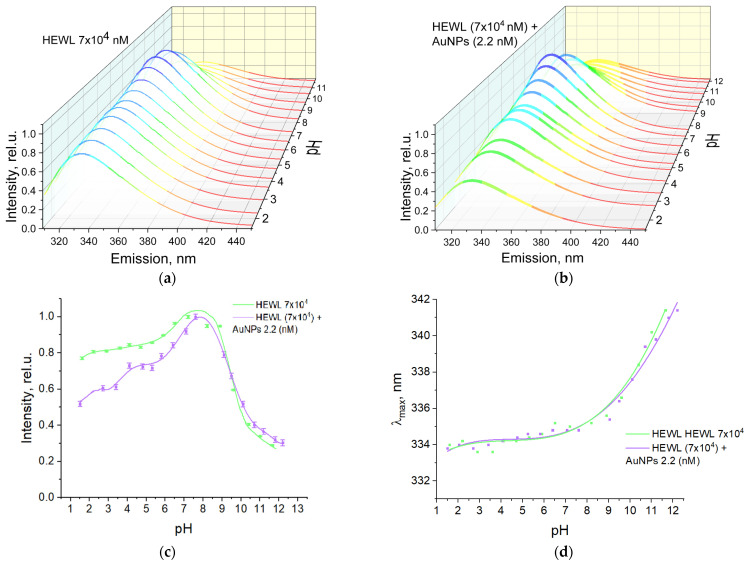
Effect of pH on the fluorescence of solutions: (**a**) HEWL (7 × 10^4^ nM) (The color of the lines reflects only the spectral range); (**b**) HEWL (7 × 10^4^ nM) + AuNPs (2.2 nM) (The color of the lines reflects only the spectral range); (**c**) Dependence of fluorescence intensity on pH of colloids HEWL (7 × 10^4^ nM) and HEWL (7 × 10^4^ nM) + AuNPs (2.2 nM); (**d**) Dependence of λ_max_ of fluorescence on pH of colloids HEWL (7 × 10^4^ nM) and HEWL (7 × 10^4^ nM) + AuNPs (2.2 nM).

The effect of pH on the fluorescence characteristics of HEWL solutions was investigated. The maximum fluorescence of the HEWL protein was observed at a wavelength of approximately 334–335 nm with an intensity of 0.8–1 rel.u. over the range of measured pH values from 1.5 to 7 (Figure 4a,c). As the pH of the medium was further increased, the wavelength of maximum fluorescence increased monotonically to 342 nm, and the intensity decreased to a value of 0.2 rel.u. When gold nanoparticles were added to the protein molecules, the maximum dependence of fluorescence intensity on pH changed (Figure 4b,d). The maximum fluorescence intensity is observed at pH 7.5. When the pH was decreased from 7.5 to 1.5, the intensity decreased from 1.0 to 0.5 rel.u., while the position of the fluorescence maximum did not change (334–335 nm). However, when the pH is increased from 7.5, the fluorescence intensity is equal to 1.0 rel.u. and decreases sharply to 0.8 rel.u. at pH = 9.0. With a further increase in pH, we observe a decrease in intensity to a value of 0.3 rel.u. The dependencies of the position of the fluorescence maximum of the protein molecules on the pH of the solution in the presence and absence of nanoparticles are quite similar. Differences are observed only at pH values below 4.0. At pH 2.0, the fluorescence intensity of protein molecules in the presence of nanoparticles is 35% lower than that of protein molecules in the absence of nanoparticles.

**Figure 5:**
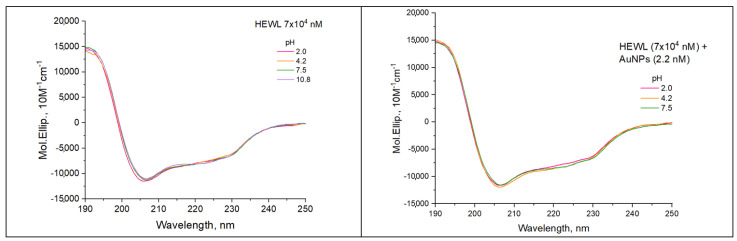
Effect of pH on the dispersion curve of circular dichroism in colloids HEWL (7 × 10^4^ nM) and HEWL (7 × 10^4^ nM) + AuNPs (2.2 nM).

The effect of pH on protein secondary structure in the absence and presence of gold nanoparticles was investigated (Figure 5, Table 1). Table 1 shows that with increasing pH, the percentage of alpha helices for the colloidal solution of lysozyme protein decreases from a value of 35.4% at pH 2.0 to a value of 32.1% at pH 10.8. The percentage of beta structures of the protein solution is maximum at pH 3.9 and is equal to 20.7%. When the pH of the initial colloid is reduced to 2.0, the beta structure content decreases to 17.8%. The content of β-sheets also decreases to 18.4 when the pH changes to the alkaline side to 10.8. HEWL structure turn content is only slightly affected by pH within 0.5%. Note also that the volume of disordered structures increases with pH above 2%.

There is an increase in the alpha-helical structures and a decrease in the beta sheets content by the values from 32.5% to 35.2% and from 20.7% to 18.7%, respectively, with the addition of gold nanoparticles to the initial colloid of HEWL at pH 3.9, shown in Table 1. For pH 3.9, there is also a change in the percentage of turns from 21.0% to 23.1% and disorder structures from 25.6% to 23.0%. After increasing the pH to 7.5, there is a slight increase in α-helices from 32.2% to 33.5% and a decrease in β-sheets from 19.6% to 18.4% compared to the protein colloid without nanoparticles at the same pH. For pH 10.8, adequate data for colloid HEWL (7 × 10^4^ nM) + AuNPs (2.2 nM) could not be obtained. It is probably because all the substrate has precipitated out.

**Figure 6:**
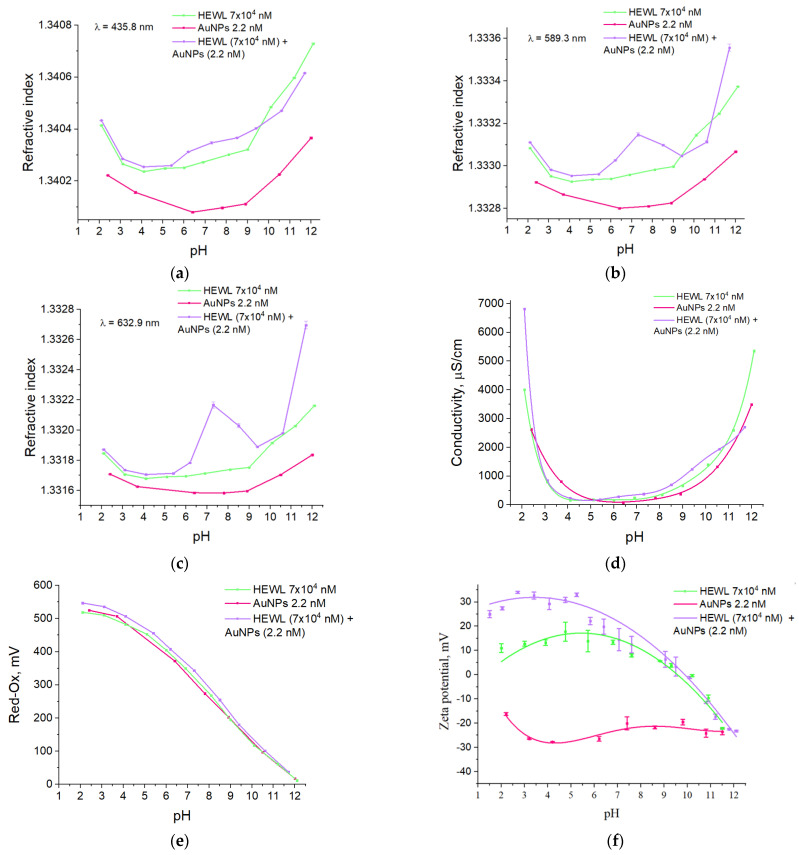
Effect of pH on the refractive index in colloids HEWL, (7 × 10^4^ nM), AuNPs (2.2 nm), HEWL (7 × 10^4^ nM) + AuNPs (2.2 nM), measured at three wavelengths: (**a**) 435.8 nm; (**b**) 589.3 nm; (**c**) 632.9 nm. Dependence of electrical conductivity (**d**), redox potential (**e**), ζ-potential (**f**) of colloids HEWL, (7 × 10^4^ nM), AuNPs (2.2 nM), and HEWL (7 × 10^4^ nM) + AuNPs (2.2 nM) from pH.

The effect of pH on the refractive index of protein solution of HEWL, AuNPs, and their mixture was studied at wavelengths 435.8 nm, 589.3 nm, and 632.8 nm (Figure 6a–c). The dependences of the refractive index on pH at all three wavelengths are similar for all groups of solutions under study. For a solution containing both gold nanoparticles and HEWL, an abrupt change in the refractive index at pH 7.3 is observed from 1.33296 to 1.33315 when measured at a wavelength of 589.3 nm (Figure 6b) and from 1.33171 to 1.33217 when measured at wavelength λ = 632.8 nm. When measured at wavelength λ = 435.8 nm, a non-monotonic but numerically smaller change in the refractive index from 1.34026 to 1.34035 is also observed.

The effect of acid and alkali additives used to change the pH on the electrical conductivity of HEWL colloids and/or AuNPs was investigated (Figure 6d). For all three colloidal systems in the pH range of 4.0–9.0, the specific electrical conductivity values remain almost unchanged (80–350 µS/cm). Transitioning to the acidic region at pH = 2.1, the electrical conductivity values increase to 2600 and 4000 µS/cm for stock solutions of gold nanoparticles and HEWL, respectively, and to 6800 µS/cm for colloid containing both gold nanoparticles and HEWL. When the medium is alkalized to pH 12.0, the conductivity values increase monotonically to 3500, 2700, and 5350 µS/cm for gold nanoparticle colloids, HEWL, and their mixture, respectively.

The effect of acid and alkali additives used to change the pH on the redox potential value of HEWL colloids and/or AuNPs was investigated (Figure 6e). When the acidity of the environment was changed in the pH range of 2.0–12.0, the redox potential of the three systems decreased almost linearly with increasing pH from 520–545 mV to 15–40 mV.

The effect of pH on the electrokinetic potential value of aqueous HEWL colloids and/or gold nanoparticles was studied (Figure 6f). The gold nanoparticle colloid has negative zeta potential over the entire pH range from −17 to −31 mV, with a weighted average value of −25 mV. The zeta potential of the aqueous HEWL colloid in the pH range of 2 to 7 has an electrokinetic potential value of the order of +10 mV. With a further increase in pH values, a decrease in the electrokinetic potential of the HEWL colloid to values of −25 mV is observed. The effect of pH on the value of the electrokinetic potential of an aqueous colloid containing both HEWL molecules and gold nanoparticles was studied. The zeta potential of the mixture of HEWL with nanoparticles in the pH range from 2 to 5 has an electrokinetic potential value of the order of +30 mV. With further increase in the pH values, a decrease in the electrokinetic potential to values of −25 mV is observed.

**Figure 7:**
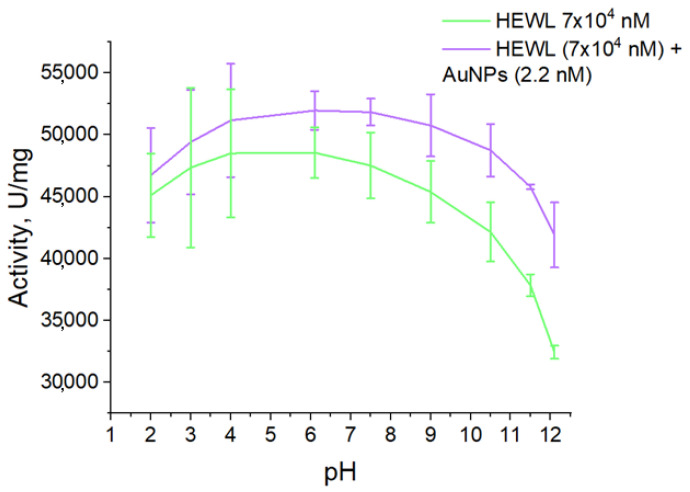
The effect of pH on the enzymatic activity of colloids HEWL (7 × 10^4^ nM) and HEWL (7 × 10^4^ nM) + AuNPs (2.2 nM).

The effect of pH on the enzymatic activity of the HEWL protein was studied (Figure 7). For HEWL, the activity values were maximum (45,000–50,000 U/mg) in the pH range from 3 to 7.5. With increasing pH, a gradual decrease in enzyme activity is observed down to a value of 31,000 U/mg. This means that the enzymatic activity of HEWL can decrease by almost 40% when the medium is alkalized. When nanoparticles are added to the HEWL colloid, there are no significant differences in the change in activity in the pH range from 2 to 6. When pH is increased to 10.5, the activity of the HEWL sample does not change significantly when nanoparticles are added and is in the range of 50,000–48,000 U/mg. When the pH is further increased to 12.1, the activity value decreases to 42,000 U/mg. This means that when gold nanoparticles are added, the enzymatic activity of HEWL does not change up to pH 10.5, and with further alkalisation of the medium, it can decrease by no more than 15%.

## 3. Discussion

Using the laser ablation method in liquid, gold nanoparticles with an average hydrodynamic diameter of about 18 nm and a half-width of about 17–20 nm were obtained (Figure 1a,b). The maximum optical density of the nanoparticle preparation is observed at a wavelength of ~520 nm (Figure 1c). In general, the spectrum corresponds to that of an aqueous colloid of small spherical gold nanoparticles [21] without any foreign chemical groups on the surface [22]. The electrokinetic potential of the gold nanoparticles was −26 mV (Figure 1d). There are cases of obtaining gold nanoparticles with almost twice the electrokinetic potential (−46 mV) using laser ablation [23]. The electrokinetic potential of the gold nanoparticles obtained in this work cannot be considered a record, although the drug should be stable for several months under normal conditions.

pH has a significant effect on the size distribution of gold nanoparticles and HEWL molecules in colloids (Figure 2). At pH above 5, dimeric forms of HEWL begin to appear in the colloid, which is in good agreement with the literature data [24]. In HEWL solution at pH above 7.5, monomeric and dimeric forms disappear, and large aggregates appear. This is probably due to the aggregation of dimers [25]. Furthermore, the addition of gold nanoparticles shifts this process on the pH scale by one–two units toward lower values. The existence of a transition process for HEWL in the presence of gold nanoparticles at an acidity of 7.5 also shows a sharp jump in the refractive index at wavelengths 589.3 and 632.9 (Figure 5). In general, the difference between the refractive indices of the HEWL + AuNPs and HEWL groups increases with increasing wavelength at which the measurement occurs. Gold nanoparticles, stable at pH 6.0–11.0, aggregate when HEWL is added to them due to the “protein corona” formed on the nanoparticles. In contrast, at pH 1.5–4.0, HEWL prevents the agglomeration of gold nanoparticles. Thus, in a colloidal system containing both HEWL and gold nanoparticles, the mutual influence of the substrates was determined.

The absorption intensity of the HEWL colloid and the protein colloid with nanoparticles does not change significantly over the whole range of pH values measured. Significant changes are only observed at the extreme pH points (less than 1.5 and more than 11.6) (Figure 3). Furthermore, at pH above 11, the protein colloid has a significantly higher optical density compared to colloids containing both protein and gold nanoparticles. The data obtained correlate well with the preservation of enzymatic activity (Figure 6). The absorption maxima of the HEWL colloid and the protein colloid with nanoparticles change symmetrically in the antiphase with changes in pH. It is known that the value of the absorption maximum depends on the size of gold nanoparticles [26]. The larger the size of the gold nanoparticles, the longer the absorption maximum shifts to the “red wavelength range” [27]. This indirectly confirms the DLS data on the size distribution of gold nanoparticles in a wide pH range (Figure 2). Namely, at pH values below 6.0, aggregation of gold nanoparticles to sizes of hundreds of nanometers occurs.

Fluorescence spectroscopy provides insight into the structural and dynamic properties of macromolecules, particularly proteins. The sensitivity of protein fluorophores to the polarity of their local environment allows for the use of fluorescence spectroscopy to analyze the conformational changes that occur in proteins under the influence of various factors [28]. The intrinsic fluorescence of proteins occurs due to the amino acids they contain, such as tyrosine, tryptophan, and phenylalanine. Tryptophan (Trp) has the highest relative absorbance compared to other aromatic amino acids, with its absorption maximum occurring at 280 nm [29]. HEWL has six Trp residues, two of which contribute significantly to the steady-state fluorescence of the protein [30]. An excitation wavelength of 280 nm was chosen to fall within the tryptophan absorption band. The maximum fluorescence intensity of the HEWL colloid and the protein colloid with nanoparticles is observed in the pH range from 7 to 8 (Figure 4). In this case, in the pH range from 7 to 8, the emission maximum begins to change from 334 nm at pH 7.5 to 341 nm at pH 12.0. It can, therefore, be assumed that for HEWL, the fluorescence intensity of protein monomers is lower than that of dimers, which is indirectly consistent with the literature data [31]. The maximum intensity is observed when protein dimers begin to lose their original conformation. During aggregation, the fluorescence intensity decreases mainly due to the screening of some molecules by others [32].

Circular dichroism provides information about the secondary structure of the protein: the number of α-helices, β-sheets, turns, and disordered structures. It is effective in the case of studying protein interactions with nanoparticles. Our colleagues have shown [33] that the size and concentration of nanoparticles affect the peptide conformation, in particular, reducing the number of α-helices and increasing the number of β-sheets. In our study, at pH 3.9 and 7.5, on the contrary, there is an increase in α-helices and a decrease in the number of β-sheets. This could probably be due to the concentration and size of nanoparticles we use and the nature of the protein.

It is known that the formation of a protein crown on nanoparticles occurs due to numerous forces, namely, van der Waals, coulomb and hydrophobic interactions, and hydrogen bonds [34]. The electrokinetic potential of gold nanoparticles is negative over a wide pH range. The protein molecules have a positive charge before reaching the isoelectric point (from 2 to 10 pH). Therefore, the main binding mechanism will be the electrostatic interaction. An increase in the value of the zeta potential when binding nanoparticles to a protein is possible due to the adsorption of a large number of positively charged protein molecules on the surface of a negatively charged nanoparticle.

Table 2 gives a visual comparison of the results obtained by optical methods for different colloids. It provides an overview of the processes described in this paper.

**Figure 8:**
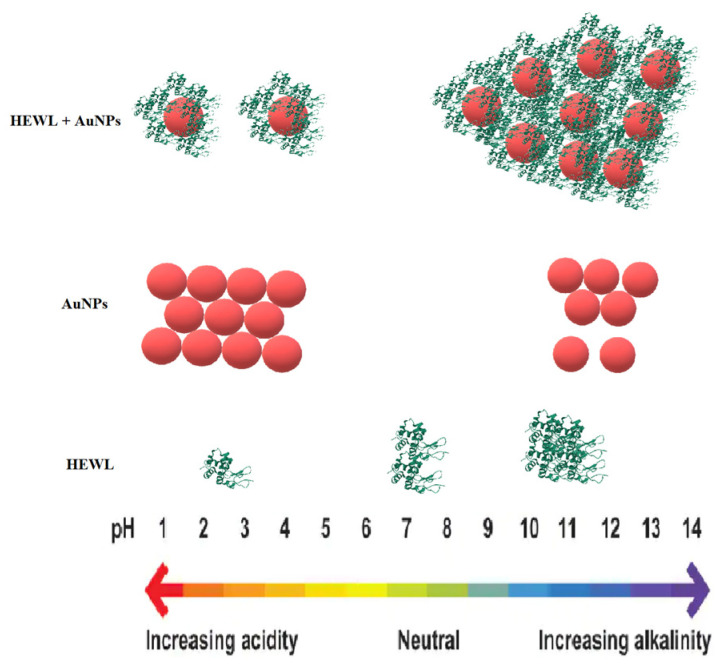
Effect of pH on colloids HEWL (7 × 10^4^ nM), AuNPs (2.2 nM), and HEWL (7 × 10^4^ nM) + AuNPs (2.2 nM).

For illustrative purposes, Figure 8 shows the results of the study of all colloids at different pH values.

## 4. Materials and Methods

Lysozyme protein molecules (HEWL, activity > 20,000 U/mg, A-3711, Applichem GmbH, Darmstadt, Germany) were used in the experiments. Lysozyme (HEWL) is one of the most accessible enzymes, as well as one of the most studied enzymes. In particular, HEWL has been used most frequently to study many aspects of protein structure and function, including stability and folding mechanisms [35]. HEWL was dissolved in deionized water to a final concentration of 7 × 10^4^ nM. Milli-Q water was used to prepare solutions; the electrical resistivity of water was ≈18 MΩ × cm.

### 4.1. Fabrication of AuNPs

Gold nanoparticles (AuNPs) are obtained by laser ablation of a massive target in deionized water. The massive target made of pure gold (99.999%) was placed at the bottom of an experimental glass cell under a 2 mm layer of deionized water. Ablation was performed using Nd:YAG laser radiation (90 J/cm^2^, wavelength 1064 nm, pulse duration 10 ns, repetition rate 8 kHz) [36]. The laser radiation was mixed over the surface of the massive target using a 2D electro-galvanic scanner (Atko, Moscow, Russia) so that each new pulse did not overlap with the previous one [37]. The morphology of the nanoparticles was studied using a Libra 200 FE HR transmission electron microscope (Carl Zeiss, Jena, Germany).

### 4.2. Dynamic Light Scattering

Hydrodynamic diameter measurements of protein molecules, AuNPs, and their aggregates were performed using a Zetasizer ULTRA Red Label (Malvern Panalytical Ltd., Malvern, UK). The experiments were carried out at 22 °C in a quartz cuvette with a sample volume of 2 mL. With all studied solutions, 3 measurements were carried out in each experiment. The measurement technique has been described in detail previously [38].

### 4.3. Fluorescence Spectroscopy

The fluorescence of aqueous solutions of HEWL molecules was studied using a Jasco FP-8300 spectrometer (JASCO Applied Sciences, Victoria, BC, Canada). Measurements were made in a quartz cuvette with an optical path length of 10 mm. Each sample was measured three times. The 2D fluorescence spectra were recorded. Measurements were performed at a temperature of 22 °C. All samples, both control and with nanoparticles, were excited at a wavelength of 280 nm, and the emission of the samples was recorded in the range of 300–450 nm; the scanning speed was 1000 nm/min. The measurement technique has been described in detail previously [39].

### 4.4. Absorbance Spectroscopy

Absorption spectra were measured on a Cintra 4040 spectrophotometer (GBC Scientific Equipment, Keysborough, Australia) in quartz cells with an optical path length of 10 mm at a temperature of 22 °C. Absorption spectra were measured in triplicate for each sample in the range 380–700 nm at a scan rate of 350 nm/min and a slit width of 1 nm. The measurement technique has been described in detail previously [40].

### 4.5. ζ-Potential Measurement

ζ-potential measurements were performed with a Malvern Zetasizer Ultra (Malvern Panalytical Ltd., Malvern, UK) at 22 °C using ZS Xplorer software (V.1.0). All measurements were performed using automatic attenuation and an automatic measurement procedure (cycle range for each measurement from 10 to 100). The pause between repetitions was 60 s, and the equilibration time was 60 s. The measurement technique has been described in detail previously [41].

### 4.6. Refractometry

Refractive index measurements were carried out using a multi-wavelength refractometer, Abbemat MW (Anton Paar, Graz, Austria). In the experiments, 1 mL of solution was poured into the cell of the device, and the measurements were carried out at wavelengths 435.8 nm, 589.3 nm, and 632.8 nm at a temperature of 22 °C. Resolution: RI ± 0.000001 nD; temperature 0.01 °C. Accuracy: RI ± 0.00004 nD; temperature ± 0.03 °C; temperature stability ± 0.002 °C.

### 4.7. Enzymatic Activity of HEWL

The enzymatic activity of HEWL was studied using *M. lysodeikticus* micrococcus cells at room temperature. An aqueous solution of lysozyme molecules, 10 μL, was diluted 100 times in 50 mM PBS buffer (pH = 7.4). The 100 µL diluted lysozyme solution was added to 2.5 mL of micrococci in 20 mM PBS pH = 7.4. In this case, the optical density at the wavelength of 450 nm was approximately 0.7–0.8. Activity was measured by the decrease in optical density on a Cintra 4040 spectrophotometer (GBC Scientific Equipment, Keysborough, Australia) during the first two minutes after the addition of lysozyme. Measurements were carried out at a temperature of 22 °C. The measurement technique was described in detail previously [42].

### 4.8. Potentiometric Studies

pH values, redox potential, and specific conductivity were measured using a Seven Excellence S470 precision station (Mettler Toledo, Greisensee, Switzerland) in 20 mL vials with constant stirring using a magnetic stirrer with a Teflon stirrer in near laminar mode. The pH of the solution was changed by adding different amounts of HCl 0.1 M (to acidify the medium) or KOH 0.1 M (to alkalize the medium). Acid and alkali were added dropwise in approximately 100 μL. The pH change step was approximately 0.5–1.0 units. The measurement technique has been described in detail previously [43].

### 4.9. Circular Dichroism Spectroscopy

Circular dichroism measurements were performed on a Jasco J-810 spectropolarimeter. Cuvette was 0.1 mm thick. The protein concentration in all colloids was 70 µM. The following three independent samples were measured. The program used for data calculation was CONTINLL (CDPro package) with the set of reference spectra SMP56. Measurements were performed at a temperature of 22 °C.

## 5. Conclusions

It has been shown that it is possible to use optical methods to monitor the change in the state of protein molecules and nanoparticles in aqueous colloids with changes in pH and to study the interaction of protein molecules with nanoparticles. Changing the pH may be the easiest way to control the protein corona on gold nanoparticles. The data obtained in the manuscript allow for the state of colloidal solutions of HEWL and AuNPs to be monitored using one or two simple and accessible methods. With all the optical methods used, a specific point of pH 7.5 was found where the transition of the colloidal system from one state to another was observed. The protective effect of gold nanoparticles on HEWL molecules at alkaline pH values is unexpected. In general, gold nanoparticles can stabilize HEWL protein molecules, which may have practical applications.

## Figures and Tables

**Table 1:** Effect of pH on the secondary structure of the protein measured by circular dichroism in colloids HEWL (7 × 10^4^ nM) and HEWL (7 × 10^4^ nM) + AuNPs (2.2 nM).

Sample	pH	Alpha Helix, %	Beta, %	Turn, %	Disorder, %
HEWL	2.0	35.4	17.8	21.1	25.7
	3.9	32.5	20.7	21.0	25.6
	7.5	32.2	19.6	21.5	26.6
	10.8	32.1	18.4	21.6	27.9
HEWL + AuNPs	2.0	35.7	17.8	21.2	25.4
	3.9	35.2	18.7	23.1	23.0
	7.5	33.5	18.4	21.1	26.9

**Table 2:** Effect of pH on the optical characteristics of colloids HEWL (7 × 10^4^ nM), AuNPs (2.2 nm), and HEWL (7 × 10^4^ nM) + AuNPs (2.2 nM).

Feature		PH	2.0	3.9	4.8	5.5	6.8	7.5	8.9	9.5	10.1	10.8	11.6
	Colloid												
Size, nm	**HEWL**		1.2 40	1.2 45	1.4 48	2.1 60	2.5 108	146	568	770	790	900	950
	**AuNPs**		1000	750	460	90	18 146	18 146	18 125	18 150	20 160	20 160	20 160
	**HEWL+** **AuNPs**		20 310	24	51	135	415	750	800	860	910	970	990
λ_max_ of absorption, nm	**HEWL**		280	281	281	280	280	280	281	281	281	280	281
	**HEWL+** **AuNPs**		279 522	280 524	279 531	280 537	281 543	280 547	280 548	280 548	280 548	281 550	281 543
	**AuNPs**		591	535	523	520	518	519	523	523	523	524	525
OD_max_	**HEWL**		2.48	2.55	2.56	2.6	2.61	2.62	2.63	2.64	2.65	2.64	2.43
	**HEWL+** **AuNPs**		2.45 0.38	2.55 0.38	2.57 0.4	2.59 0.4	2.6 0.39	2.54 0.31	2.55 0.30	2.55 0.29	2.53 0.26	2.49 0.26	2.3 0.24
	**AuNPs**		0.18	0.32	0.42	0.45	0.48	0.47	0.43	0.40	0.37	0.34	0.33
λ_max_ of fluorescence, nm	**HEWL**		334.2	333.6	334.2	334.4	335.2	335.2	335.6	336.6	338.2	340.0	341.4
	**HEWL+** **AuNPs**		334.0	334.2	334.4	334.6	334.8	334.8	335.4	337.6	337.6	339.4	341.0
Fluorescence intensity, rel.u.	**HEWL**		0.78	0.82	0.83	0.87	0.97	1.0	0.94	0.6	0.45	0.37	0.29
	**HEWL+** **AuNPs**		0.53	0.70	0.72	0.75	0.88	1.0	0.82	0.67	0.52	0.40	0.34

## Data Availability

Data are available on request due to restrictions, e.g., privacy or ethics.
